# The Murine Lung Microbiome Changes During Lung Inflammation and Intranasal Vancomycin Treatment

**DOI:** 10.2174/1874285801509010167

**Published:** 2015-11-03

**Authors:** Kenneth Klingenberg Barfod, Katleen Vrankx, Hengameh Chloé Mirsepasi-Lauridsen, Jitka Stilund Hansen, Karin Sørig Hougaard, Søren Thor Larsen, Arthur C. Ouwenhand, Karen Angeliki Krogfelt

**Affiliations:** 1 National Research Centre for the Working Environment, Lersø parkallé 105, 2100 Denmark;; 2 Applied Maths, Keistraat 120, 9830 Sint-Martens-Latem, Belgium; 3 Statens Serum Institut, Artillerivej 5, 2300 Denmark;; 4 Active Nutrition, Dupont Nutrition & Health, Sokeritehtaantie 20, 02460 Kantvik Finland

**Keywords:** Antibiotic, broncho-alveolar lavage, carbon nanotubes, Denaturing gradient gel electrophoresis, probiotic

## Abstract

Most microbiome research related to airway diseases has focused on the gut microbiome. This is despite advances in culture independent microbial identification techniques revealing that even healthy lungs possess a unique dynamic microbiome. This conceptual change raises the question; if lung diseases could be causally linked to local dysbiosis of the local lung microbiota. Here, we manipulate the murine lung and gut microbiome, in order to show that the lung microbiota can be changed experimentally. We have used four different approaches: lung inflammation by exposure to carbon nano-tube particles, oral probiotics and oral or intranasal exposure to the antibiotic vancomycin. Bacterial DNA was extracted from broncho-alveolar and nasal lavage fluids, caecum samples and compared by DGGE. Our results show that: the lung microbiota is sex dependent and not just a reflection of the gut microbiota, and that induced inflammation can change lung microbiota. This change is not transferred to offspring. Oral probiotics in adult mice do not change lung microbiome detectible by DGGE. Nasal vancomycin can change the lung microbiome preferentially, while oral exposure does not. These observations should be considered in future studies of the causal relationship between lung microbiota and lung diseases.

## INTRODUCTION

Novel culture independent techniques for microbial identification have within a decade changed the way we view the microbiome that inhabits every crevasse of our bodies. Compartments previously considered sterile seem to be colonized and hosts unique microbiomes. The surfaces area of the adult human lungs is similar to that of the intestine or approximately 30 times, that of the skin [1, 2]. And even so, most of the microbiome research has focused on the gut microbiome and its relation to diseases. Recently it has been shown that also airways include their own complex communities of bacteria in both healthy and diseased lungs [3-5]. This warrants an investigation, whether microbiome related lung diseases could be a result of the local dysbiosis of the lung microbiota (LM) and not only from the gut microbiota (GM). The putative role of the LM and the metagenomics in the development of lung diseases, although in its infancy, is a rapidly growing field of research [6-9]. The biggest challenge now, in our opinion, is to move from correlative and descriptive studies to more functional and hypotheses driven experimental research in lung microbial ecology. Due to technical and ethical restrictions mechanistic questions can often not be studied in humans and approaches using animal models are therefore required. 

We have previously published the first description of the bacterial LM in mice using 16S rRNA gene sequencing. Bacterial communities from broncho-alveolar lavage fluids (BAL) and lung tissue were compared to samples taken from caecum and vaginal lavage fluid from female BALB/cJ mice. The naïve female mouse LM is dominated by *Proteobacteria, Firmicutes, Actinobacteria, Bacteroidetes *and* Cyanobacteria* [10].

In the present study, we investigate the possibility of manipulating LM in two different mouse strains BALB/cj and C57BL/6. If local changes to LM and not only GM play a part in the development of airway diseases, it is important to show that the LM can be changed by manipulating its genetic, chemical or immune determined ecological niche. The reasoning is that LM composition needs to be sensitive to stimuli in order to have a putative role in disease development and that new tools are needed for the study of lung microbial ecology. 

### Changes in Lung Microbiota after Stimuli

To describe changes to LM after stimuli, we performed denaturing gradient gel electrophoresis (DGGE) analysis on the bacterial 16S rRNA genotype profiles of airway and GM after exposure or mock exposure of mice and in some experiments also their offspring. DNA was extracted from BAL and from caecum samples. The DGGE method is extensively used to compare gut bacterial profiles without preexisting knowledge of their composition using universal primers based on 16s RNA gene sequences [11-13]. 

Here, we show that the established DGGE method can also be used to address diversity shifts lung microbiota. The aim of this study was to examine if LM can be changed by particle induced lung inflammation, oral intake of probiotics or exposure to antibiotics either by the airway or oral route. The exposure types were chosen for their known capabilities to change GM and their hypothetical likelihood to induce changes to LM. 

### Nano Particle-induced Lung Inflammation

Prenatal exposures to cigarette smoke, air pollution and particles cause lung inflammation that may affect immune development and increase allergic responses [14-16]. We have induced lung inflammation in mice prior to mating by intratracheal instillation with carbon nanotubes (CNT) and subsequently studied changes in lung microbiota of exposed dams, PBS controls and respective offspring. Our hypothesis is that the maternal (CNT) exposure and resulting airway inflammation would induce changes to lung microbiota in the adult mice that could possibly be transferred to the offspring. The hypothesis is that prolonged inflammation would lead to changes to the lung as an ecological niche and thereby infer a change to any active live LM. Since we can only speculate on the origin of the LM, we hypothesized that changing the lung environment and LM of the mothers would influence the LM of the offspring as well.

### Influence of Oral Probiotics on Lung Microbiota

Probiotics are “live microorganisms that when administered in adequate amounts, confer a health benefit on the host". With growing appreciation for the importance of a healthy GM, there is increasing interest in using probiotics to modulate GM in the prevention of airway inflammatory diseases such as asthma and rhinitis [17, 18]. Human studies show that administration of probiotics during pregnancy or in early life influences GM composition and possibly disease development [19-23]. Animal experiments have also identified important immune-regulatory effects of GM related to lung diseases [24-26]. Our hypothesis is that orally administered probiotics could also change the LM by colonization in the lung or indirectly by immune-stimulatory systematic effects. In the present study we have fed adult mice with different commercially available probiotics and investigated changes to GM and LM.

### Antibiotics

Whether antibiotics are involved directly in the development of asthma is subject to debate and has been extensively reviewed [27-29]. However, antibiotic administration is widely used as a means to manipulate microbiota in mouse models of disease [30-33]. Vancomycin is a glycopeptide that targets Gram positive bacteria and can alter the outcome of murine experimental allergic asthma. When vancomycin is administered orally in clinical relevant doses throughout life and in shorter perinatal exposures, it can directly change immune responses and GM in a C57BL/6J mouse model [30, 34]. We have chosen vancomycin because it is used in the treatment of human disease and because it is poorly absorbed into the body when given orally [35]. This will supposedly allow us to manipulate the GM in the gastrointestinal tract avoiding lateral systemic effects. Vancomycin is currently also undergoing clinical trials for delivery through the airways to CF patients (http://clinicaltrials.gov/ show/NCT01509339). In the present study we have exposed adult female mice either through drinking water or the airways. By dosing the mice with vancomycin via nasal instillation, we hoped to manipulate the LM preferentially to the GM. We are unaware of any studies describing systemic absorption of vancomycin from off-lable inhalations therapies.

## MATERIALS AND METHODS

### Animals and Sample Collection

Animals used in this study are BALB/cJ or C57Bl/6J mice, (Taconic M&B, Ry, Denmark), 7-8 weeks old, body weight 18-22 g. All animals within a study were bought from the same breeding lot as non-siblings. Animals were randomly distributed and housed 10 (BALB/cJ) or one (C57Bl/6J) for breeding animals per cage (425×266×150 mm) with tap water and food (Altromin no 1324 Brogaard Denmark) provided *ad libitum*. Light /dark cycles were at 12 hours and room temperature and relative humidity was kept at 19-22ºC and 40-60%, respectively. Animals were handled by the same two animal facility caretakers and acclimatised in our animal facility for one or two weeks before use. 

The BAL procedure was performed as previously described [10]. Nasal lavage (NAL) was performed by inserting a sterile tube (Insyte, BD, Denmark) through the trachea into the nasal cavity and flushing once with 1.0 mL pyrogenfree saline (0.9%) (Fresenius Kabi, Denmark) into a sterile tube from both nares. All the BAL and NAL samples were frozen at -80 ºC until further processing. Caecum samples were excised using sterile scissors where after approximately 50 mg stool was removed using sterile disposable plastic loops directly into cryo tubes and snap frozen in liquid nitrogen. 

All experiments are in accordance with Council of Europe Convention European Treaty series 123 and the Danish Animal Experimentation Act (LBK 1306 of 11.21.2007). All protocols and procedures were approved by the Danish Animal Experiments Inspectorate procedures and were performed in accordance with the standards set forth in the eighth edition of Guide for the Care and Use of Laboratory Animals. Data are reported according to the ARRIVE guidelines for reporting in *in vivo* experiments [36] 

### Naïve Mice

BAL, and caecum samples were compared by DGGE to visualize differences between LM and GM in 9 week old naïve BALB/c females (n=10) and represents a single experiment.

### Prenatal Exposure to CNT Particles

C57Bl/6 mice, were anesthetised by isoflurane and instilled through the trachea (i.t) with 67 µg CNT (NM-400) (n=10) in PBS or sterile PBS (N=10) i.t. the day before mating, representing a single experiment. This dose and preparation have previously been validated by our lab to give prolonged lung inflammation [37]. At weaning on postnatal day 21 the pups were removed to separate cages and the adult dam was killed. One male and one female offspring from each dam (cages) were killed before reaching sexual maturity at 6 weeks of age. Only 1 male and 1 female offspring from the same mother were used to avoid litter and cage effects. At weaning of the pups, BAL cell composition in dams was determined as previously described [38] by flushing the lungs 2 times with 0.8 mL saline and collecting cells on cytospin slides, which were stained with May-Grüneald/Giemsa followed by cell differentiation. Neutrophil granulocyte influx was used to assess lung inflammation after pulmonary exposure. DGGE bacterial profiles of LM were compared between CNT- and PBS-exposed dams and also between their offspring (N=5 male and 5 female offspring per group).

### Probiotic Feeding

Female BALB/cJ mice were randomly assigned to cages (n=7) and conditioned to the stable environment for 14 days. For probiotic dosing, they were moved to separate clean cages alone and fed one half Oats Cheerios Breakfast cereal (Nestlé, Denmark) soaked in either PBS or 200µl probiotic suspension of a single strain and returned to their littermates exposed to the same probiotic. The enoculated cereal was dropped into the cage and eating was observed. The entire inoculum was eaten within seconds never missing one feeding. The mice received 10E9 CFU/day or PBS for 2×5 days. Two days after the last exposure, the animals were killed and BAL and caecum samples were collected. Data represent a single experiment

Three different bacterial strains were chosen for this study: *Lactobacillus acidophilus* NCFM (ATCC 700396), *Bifidobacterium animalis *ssp.* lactis* Bl-04 (ATCC SD5219) and *Bifidobacterium animalis *ssp.* lactis* Bi-07 (ATCC SD5220), all from DuPont Nutrition & Health, Finland. These strains are under interest for use in asthma and allergy treatments and as well as reduction of respiratory tract infection risk, and have been extensively used in animal models and clinical trials [39, 18, 40, 41]. Bacteria were grown in overnight cultures in 14 mLl liquid MRS /(0.5g cystein-HCL/L)- growth media at 37°C without stirring (MRS Broth, OXOID , Merck, Denmark). Before use they were harvested and spin washed in PBS and re-suspended in 0.5mL PBS. The bacteria were checked by microscopy and colony forming units (CFU) were determined on solid MRS AGAR plates (cystein 0.5g/L) after overnight growth under anaerobic conditions using AnaeroGen and jar (Thermo Scientific, Denmark).

### Antibiotic Treatment 

After two weeks of acclimatization in group specific cages, female BALB/cJ mice were exposed either to oral (O) or intranasal (IN) vancomycin or intranasal PBS (PBS). The O group (n=7) was given 200 mg/L vancomycin (Fresenius Kabi, Denmark) *ad libitum* in drinking water from a dark water bottle ad libitum for 14 days [30]. The vancomycin-water was changed every two days. The IN (n=8) and PBS (n=6) groups were anesthetised by isoflurane and dosed with 35 µL for 2×5 days with two days in between covering both nares by intra nasal instillation (i.n.). The IN groups were dosed with 0.12 mg vancomycin and the PBS group with pyrogen free saline (0.9%) (Fresenius Kabi, Denmark). Two days after the last exposure, the animals were killed and BAL, NAL and caecum samples were collected. Data represent a single experiment.

### DNA Extractions, PCR and DGGE 

DNA extractions from frozen BAL and NAL samples were done using Qiagen spin protocol (Qiagen, DNA mini kit Denmark) and (Qiagen, DNA mini stool kit Denmark) for detection of pathogens in frozen stool samples , with minor modifications as previously described [10]. We included an extraction contamination control, by performing mock extractions on our stock saline. The DGGE-PCR amplifying the V2-V3 region of the 16S ribosomal DNA gene of the bacteria was amplified by universal primer HDA1 and HDA2. The primer set HDA 1-GCf: (5´ACT CCT ACG GGA GGC AGC AGT´3) and HDA 2r (5´GTA TTA CCG CGG CTG CTG GCA C´3). The forward primer HDA 1-GCf: was labelled at the 5´ end with a GC clamp (5’-CGC CCG GGG CGC GCC CCG GGC GGG GCG GGG GCA CGG GGG -3’)” [11]. Total PCR volume of 50 µl contained: 4 µl Milli Q water, 20 µl of 5 PRIME Mastermix (MasterMix-100Rxns, 5PRIME GmbH, Hamburg), 8 µl of primer HDA 1f, 8 µl of primer HDA 2r, and finally 10 µl of DNA template. The PCR was performed under the following conditions: denaturing was carried out at 94°C for 4 min., followed by the denaturing step 94°C for 30 sec., then 30 cycles with annealing step at “56°C for 30 sec., extension at 68°C for 60 sec.”, and the final extension step performed at 68°C for 7 min. The PCR fragments were separated by DGGE as described with minor modifications including 3 lanes of marker and loading one no template PCR control per run [12, 42]. Gels were run at 60°C for 16 h at a constant voltage of 70 V in 0.5 × TAE buffer and stained with Gel-Red (Thermo Scientific, Denmark) for 60 min and photographed on the Gel Doc system (Biorad, Sweden).

### Data Analysis

Analysis of the DGGE was performed blinded using BioNumerics Version 7.5 (Applied Maths NV, Sint-Martens-Latem, Belgium). After normalization, a band matching was performed with a tolerance of 1% and an optimization of 1%. The results from the automatic band matching were checked manually and corrected where necessary. The similarity between profiles was calculated with a ranked Pearson correlation coefficient based on the band matching results using band intensities. A dendrogram was then constructed with UPGMA. The reliability of the dendrogram was determined with a cophenetic correlation coefficient [43]. For this, you look at the cophenetic correlation on the dendrogram. Reliably seperated branches have a high cophenetic. For each profile, the Simpson’s index of diversity and Pielou species evenness were calculated [44, 45]. All statistical analyses were performed after mean-based normalisation of profiles. For the comparison of two groups, t-test was used, for multiple groups analysis of variance (Anova) with post-hoc test Bonferroni. The level of significance was set at P < 0.05.

Inflammation in dams in the prenatal exposure to CNT particles was determined by BAL cell composition (Epithelia, neutrophil and eosinophil- cells) with parametric (ANOVA) and non-parametric (Kruskal-Wallis) analysis.

## RESULTS 

### DGGE on Naïve Mice

Comparing BAL and caecum samples taken from 9 week old naïve BALB/c females (n=10) the results show a clear separation of the two microbiomes. As seen from Fig. (**1a**) the LM samples from BAL form a clearly delineated cluster separate from the corresponding GM from caecum samples. The caecum samples have as expected a much higher diversity with less similarity between mice than the LM samples. The sample clustering is also evident on a PCA plot (Fig. **1b**) where the LM samples form a tight separate cluster.

### Nano Particle-induced Inflammation

After birth and weaning of their pups, the now 14 weeks old C57Bl/6 dams were killed and LM was compared between the CNT (n=10) or PBS (n=10) dams. Based on differences in cell types and especially neutrophil composition, the lung inflammation of the CNT group compared to the PBS groups was statistically significant (p = 0.004). The internal bacterial clustering between samples is clear at the dendrogram in Fig. (**2a**). Visualized in the PCA plot in Fig. (**2b**), the PBS samples cluster together and cluster with some of the CNT samples. Some samples from the CNT group are outliers, these outliers contained additional bands that were either not present or much weaker in the other profiles. Even so, there is neither statistically significant difference between the average diversity nor the average species evenness of LM from CNT and PBS dams. We did try to correlate these outliers with disease/ inflammation severity based on number of inflammatory cells both neutrophils and eosinophils. These correlations were not statistically significant.

Offspring from both maternal exposure groups were killed before reaching fertility, at the age of 6 weeks. As seen in Fig. (**2c**) the offspring DGGE profiles of LM shows no correlation to the dam´s exposure. No statistical significant differences are seen between CNT and PBS, neither in the whole group or in only the males or females. By serendipity, there was a clear difference in LM based on sex seen as two clear clusters. There are 4 bands statistically significantly different between the males and females and it is a true absence/presence difference. This is evident both in Fig. (**2c**) and (**2d**), where males and females cluster separately from each other.

### Influence by Oral Probiotics Onlung Microbiota

After 2 weeks of feeding with probiotic or PBS and two days after last exposure the now 11weeks old female BALB/cJ mice (n= 28) were killed. Airway microbiota from NAL (NM) and BAL (LM) were compared between the probiotic and PBS control groups. Due to the high number of samples they were distributed between DGGE runs in an ordered random fashion to minimize the effect of variation between DGGE runs. Two samples were removed from the analysis due to a loading error. No apparent clustering was observed corresponding to any of the parameters in the dendrograms or PCA –plots (data not shown). For most mice, the NM and LM from the same mouse were found in the same cluster, but this was not the case for all mice. The conclusion from dendrograms and PCA plots was the same. It was not possible to distinguish between NM and LM within or across exposure groups.

No distinct band corresponded to the band size of the probiotic strain used as a positive DGGE-PCR control in the study in each exposure group. To further explore the data, we performed a statistical analysis to check if any bands were stronger or weaker in any of the probiotic groups. No significant differences were seen with mean centered normalisation and Anova.

### Antibiotic Treatment

When comparing GM between the three exposure groups oral (O) or intra nasal (IN) vancomycin or intra nasal PBS (PBS), the O-group presents, as expected, with an altered GM compared to the other two groups. The exposure groups were housed with same type exposure animals, excluding any possibility of cross contamination. Fig. (**3a** and **3b**) show that the GM from all animals in the O- group cluster separately, except for 1 sample. This corresponds well visually during necroscopy, where the O-groups had very loose fecal matter. The statistically difference is significant but weak since there is only one band that is significantly different and more intense in the O- group below the lowest marker band. There is one band that is significantly different with Anova between the three groups. This band has the highest average intensity in the O-group and is marked with a black box on Fig. (**3a**). However, this band has low intensity and below the marker, in the less reliable part of the gel. More data are needed to determine whether this difference corresponds the result of a true biological difference. Intranasal exposure to vancomycin does not influence GM as compared to PBS. 

When comparing airway (BAL and NAL) samples from all three exposure groups we could not distinguish between NM or LM. No clustering was shown between samples from the upper respiratory tract NM or the lower from BAL (LM) (results not shown). Fig. (**3c** and **3d**) shows the comparison between exposure groups for BAL samples only. The clustering indicates that intranasal instillation of vancomycin changed the LM of the IN-group compared to intranasal PBS or oral Vancomycin but there are no statistical differences between the groups. The band with the lowest p-value (0.088) was the left most band. The samples were distributed between DGGE gels and runs in an ordered random fashion to minimize variation between runs.

## DISCUSSION

The human upper respiratory tract is known to house an abundant community of microbes, but the healthy human lung has long been considered to be free of bacteria. This notion stems from our definition of bacterial presence, which previously was based on our ability to cultivate bacteria under laboratory conditions. Novel culture independent techniques such as DGGE or next generation sequencing of bacterial 16SrRNA-gene (NGS) for microbial identification have within a decade changed our view on bacterial presence. The current conceptual challenges are to identify; what part of the microbial signals detected in the lower respiratory tract in human samples is actually a true microbiome. The alternative explanation for the compiling evidence on bacteria in the human lungs is that they are artifacts from sample acquisition, DNA extraction or occur due to NGS methodology issues [46]. When performing DGGE on mock extraction samples we do see 3-4 faint bands. These bands disappear when any template DNA is added and the bands are thus not present in any samples. Perhaps lung microbiota data simply reflects what is currently inhaled in a state of flux within the local environment. It is still not fully disclosed from where we obtain our LM, [47] but to approach a new conceptual model of lung pathogenesis, it is imperative to investigate whether LM can change with stimuli in its host genetic, chemical or immune environment. In mice, it has been shown that the GM changes with a range of environmental or introduced factors such as parental lineages, animal caretakers, mode of delivery, stress or pro- or antibiotics [26, 48, 49, 50].

### Naïve Mice 

In the study on naïve mice, we have shown that the LM is not just a reflection of the GM at a given time point. We hypothesized that if the LM was just a reflection of the local environment in their litter boxes then the LM would have a huge overlap with the GM, due to fecal matter in the box. The DGGE results confirm that the LM is indeed different from the GM. This finding therefore corroborates our previous report using NGS [10]. The DGGE pattern of LM shows low variation between individual mice. Barring methodology issues, this either suggests that all the LM from the different mice stems from a very similar airway exposure to an unknown bacterial background different from that of the GM. Or that LM is similar between mice because equal ecological biological niches in the lungs shape the microbiota. A limit to this first study was the lack of samples from NAL representing the upper respiratory tract. NAL samples were therefore included in some later studies. 

### Prenatal Exposure to CNT Particles

We investigated whether or not particle-induced airway inflammation could change LM, as investigated by DGGE. We expected a very clear difference in LM between the mice with prolonged airway inflammation compared to the PBS group (Supplementary Fig. **1**) due to the radical change in the lung environment as an ecological niche for living LM. Although the changes were visible in several CNT DGGE profiles the overall difference between groups did not acquire statistical significance. Four CNT outliers shared one particular intense band not found in the other samples. This indicates an imbalance in the microbial community leading to one or two species dominating the population (Fig. **2a**). It is possible, that the time between exposure to CNT and the BAL procedure of approximately 6 weeks enabled the lung inflammation to progress differently between the exposed mice. At necroscopy, 6 weeks after instillation there was large variation in the lung inflammation as by judged by coloration and visible particles of the adults. This could be caused by variations in particle instillation and clearing over time. For future research it would be interesting to address microbiota changes under both short and long-term exposure scenarios. In the offspring, as expected, no visible inflammation or CNT particle patches were observed by necroscopy and no DGGE clustering based on parental exposure was shown. By serendipity, a very clear result was an observed sex dependence of LM. It is known that there are sex dependent differences in GM [51, 52] but this is for the first time being shown for LM. The sex difference in LM also goes a long way to show that the DGGE profiles for LM are not a result of background contaminations or methodological issues. These mice have lived with their siblings in the same boxes inhaling the same background air and the DNA has been extracted with the same kits and procedures. It would be very interesting to further investigate how the underlying sex difference manifests at species level with NGS. This observation also warrants the speculation that any impact LM has on lung disease etiology might be sex dependent. Almost all diseases have sex dependent traits, but it is worth noting in this context, that childhood asthma is much more frequent in boys than girls [53]. 

### Probiotic Feeding

In the probiotic part of this study we hypothesized that oral supplement with probiotics would change the composition of the LM. This could be directly by colonization or indirectly by impacting the immune system through the gut. DGGE did not indicate any change in LM or the upper respiratory tract through NAL samples, based on diet supplementation. This suggests that oral probiotics do not affect the LM. There are however several limitations to this study. First of all these experiments were done only in adult female mice. To maximize the possibility of changing LM by oral probiotics, it is very likely that this should be attempted in the same “window of opportunity” in early life as known from the succession of GM [54-56]. In a recent study, colonization of the lungs in a GF mouse model with live *Lactobacillus spp.* indicated an impact on lung development [57]. In our study we have used overnight grown live probiotic strains. It might be better to mimic the human condition and the humans study reports and use freeze dried probiotic products. It depends on how we expect humans to be exposed to the probiotic via a supplement (pill) or through functional foods such as probiotic yoghurt. Also, we have only used one doses-feeding scheme, which could be changed allowing for a longer or larger exposure. But compared to the human trials the mice in this study did receive rather high doses. It could also be that any changes are not affecting the most dominant species and therefore not visible by the DGGE method. Future investigations could attempt direct nasal instillation of probiotics or to use more sensitive methods of phylum specific 16SrRNA-gene QPCR or NGS. 

### Antibiotic Treatment

In this part of the study we exposed adult female mice either through drinking water or the airways. In order to perform experiments to assess the LM´s influence on lung immune development and disease, we considered it essential to be able to manipulate the LM separately from the GM. Most microbiota research in regards to lung diseases have focused on the influence on the lung by a changed GM. Although several studies can show an effect on experimental airway allergies with the manipulation of GM very few have even considered investigating the influence from changes in LM. Most of the experimental setups cannot distinguish whether the effect comes from changed GM or LM making results hard to interpret and compare. 

Our results suggest that by dosing mice with vancomycin via the airways, we can manipulate the LM preferentially, without a large impact on GM. Even though some of the antibiotics will be swallowed after intra nasal exposure and thus may affect the GM, the DGGE profiles of GM the IN groups is not different from the i.n. PBS groups. The doses of vancomycin given to the orally exposed mice through the drinking water must be considered (by calculation) to be much higher than the nasal instillations. Nevertheless, the LM of the O-group matches the one found in the i.n. PBS group, with only the IN group clustering separately. It is very likely that we would have a stronger signal and clustering with larger experimental groups (n=10). If i.n. vancomycin can change the local LM indicates that the LM is susceptible to antibiotics, only effective on active and propagating bacteria. This adds to the notion, that the observed LM is not just bacterial DNA debris originating from background or methodological issues, but consists of live susceptible bacteria. Although it could be indirect effect of the drug aspiration since inhaled antibiotics could have other biological effects such as renal effects known from intravenous vancomycin in humans.

The experiments were only performed with female animals except for the offspring in the particle exposure study. We show that there is a sex dependent difference in the LM. This should be taken into account when designing future investigations of LM and GM. Although it is unknown whether there are differences between the upper respiratory tract and the lower respiratory tract in mice, the NAL/BAL comparison was inconclusive probably due to the low DNA yield from NAL. NAL might also not be the best not representative of upper respiratory tract and in the future one should consider an oral specimen, either by rinse or swab or tongue homogenate. It will be necessary to use a more sensitive method like NGS or change DNA extraction strategy to investigate the possible difference between the upper respiratory tract and the lower respiratory as it is known from the human lung.

## CONCLUSION

Most experimental studies on the microbiome and its relation to airway disease focus on the influence from the gut microbiota on immunity and disease. It is a valid hypothesis, that most of the immune and health effects in the lung contributed to dysbiosis with GM, could in fact with fewer assumptions be a result of changes in the local LM instead or both. It is worth noting that so far no investigation into LM with DGGE or 16S rRNA-gene sequencing in humans or animals showing total sterility has been put forth. We show that it is possible to use DGGE-16S rRNA-PCR for the study of lung microbiota. We used it to show that the LM is different from GM, is sex dependent and can be altered during induced inflammation or by intranasal instillation of antibiotics. We suggest that DGGE should be used in tandem with NGS improving on the future use of NGS in model systems. Our observations on LM could possibly be used in future investigations of the LM putative causal role in health and disease and challenges the traditional models of pathogenesis in infections and the development of chronic inflammatory diseases such as asthma and COPD.

## Figures and Tables

**Fig. (1a):**
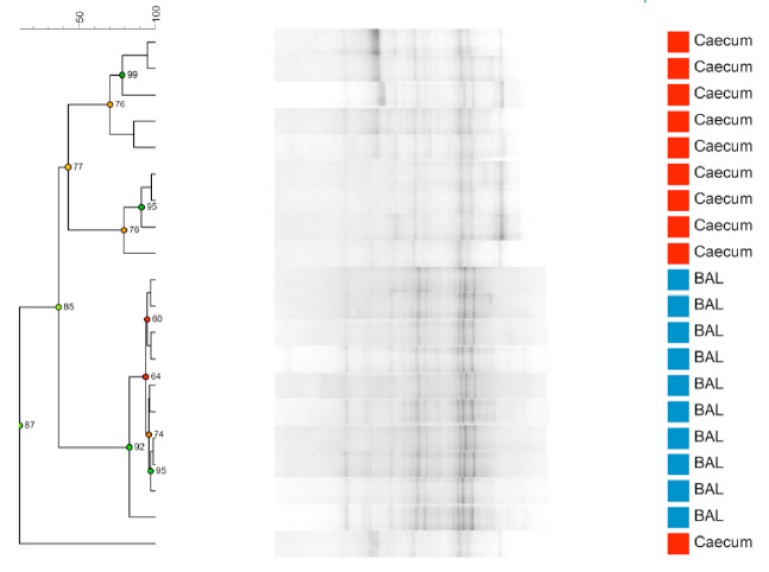
Dendrogram based on DGGE profiles representing 16S rRNA gene PCR-derived amplicons of blue BAL and red caecum samples collected from nine-weeks-old naïve female BALB/cj (n=10). DGGE: the BAL samples form a tight cluster that can be distinguished clearly from the caecum samples. Reliably separated branches on the dendrogram have a high (green) cophenetic score.

**Fig. (1b):**
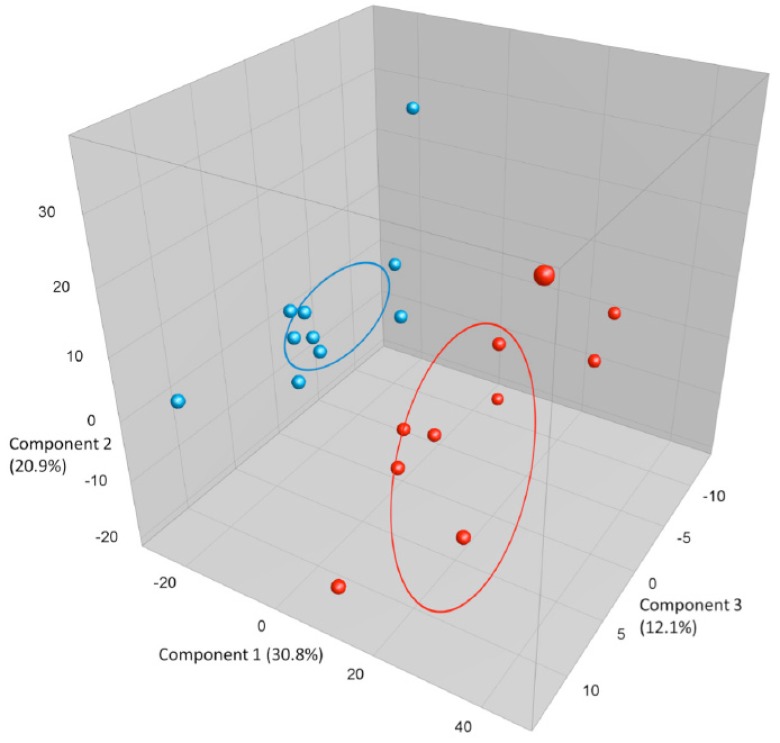
PCA plots based on DGGE profiles of 16S rRNA gene PCR-derived amplicons of blue BAL and red caecum samples collected from nine-weeks-old naïve female BALB/cj (n=10). DGGE: denaturing gradient gel electrophoresis after DGGE-PCR: polymerase chain reaction. There is a group to the left that consists of all BAL samples clustering separately from the caecum. The variation between the BAL samples is much lower than between the caecum samples.

**Fig. (2a):**
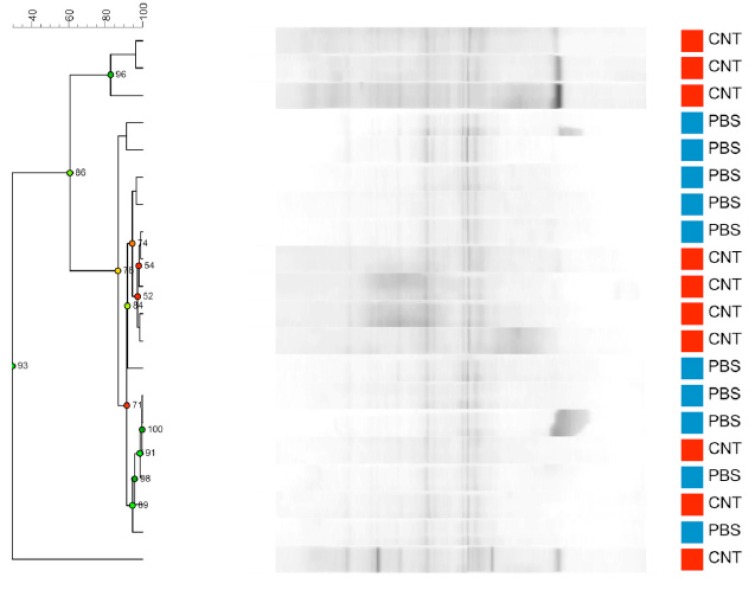
Dendrogram based on DGGE profiles representing 16S rRNA gene PCR-derived amplicons of BAL samples collected from C57Bl/6 dams exposed i.n. to either PBS (blue) or CNT particles (red) before mating (n=20). Most samples are very similar, but a few samples have intense bands that are absent or weaker in the remaining samples. These samples all belong to the 4 defining CNT samples from the CNT group. Reliably separated branches on the dendrogram have a high (green) cophenetic score.

**Fig. (2b):**
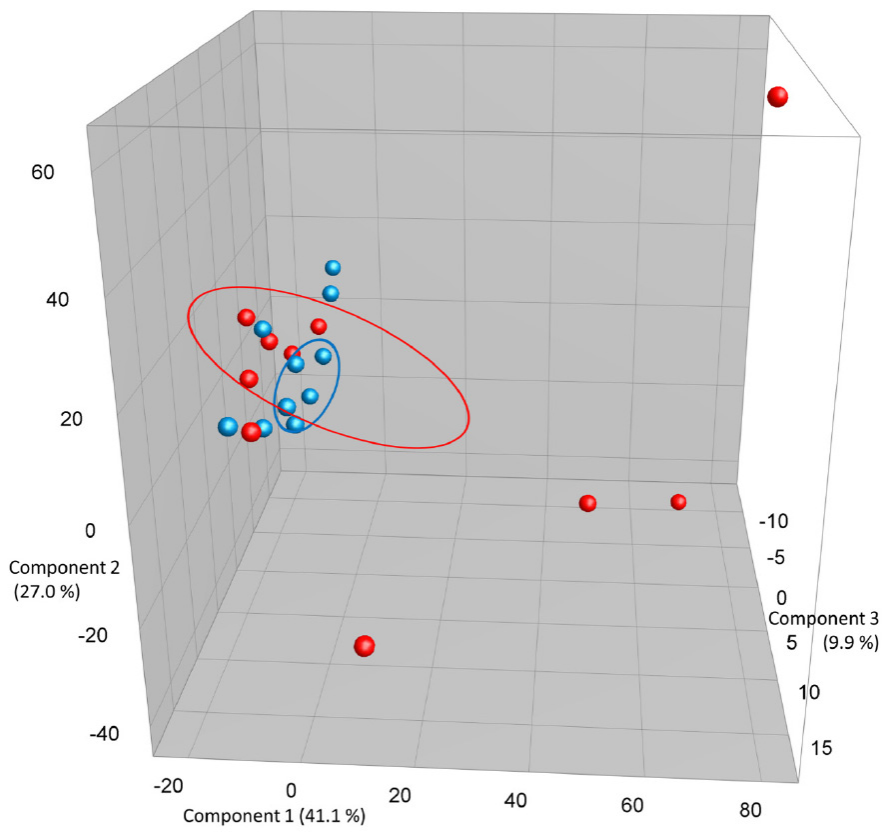
PCA plots based on DGGE profiles of 16S rRNA gene PCR-derived amplicons of BAL samples collected from C57Bl/6 dams exposed i.n. to either PBS or CNT particles. The colors represent red: CNT dams and blue PBS dams (n=20). DGGE: denaturing gradient gel electrophoresis after DGGE-PCR: polymerase chain reaction. There is a group to the lower left that consists of both control and CNT dams, but the outliers that cluster separately are all CNT.

**Fig. (2c):**
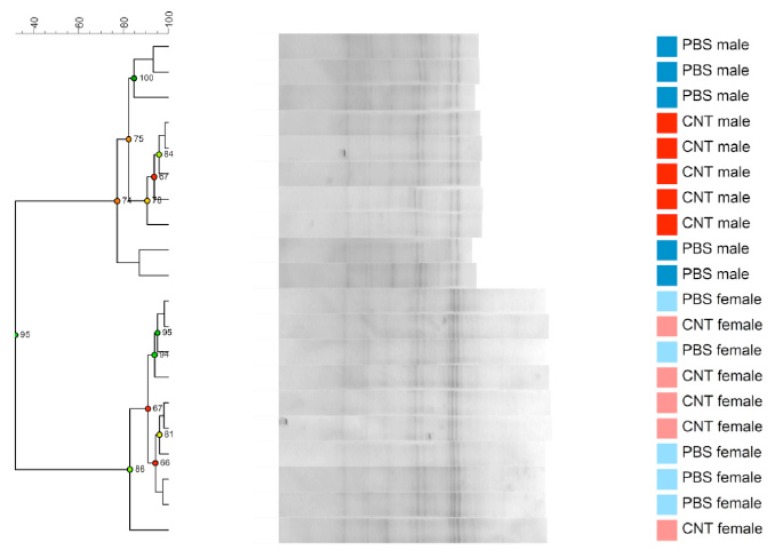
Dendrogram based on DGGE profiles representing 16S rRNA gene PCR-derived amplicons of BAL samples collected from 6 weeks old C57Bl/6 mice. The mice are both naïve male and female offspring, from C57Bl/6 dams exposed i.n. to either PBS or CNT particles before mating. The colors represent red: CNT-male, pink CNT-female and dark blue PBS-male and light- blue PBS –female.(n=20). Males and females clearly cluster separately. Reliably separated branches on the dendrogram have a high (green) cophenetic score.

**Fig. (2d):**
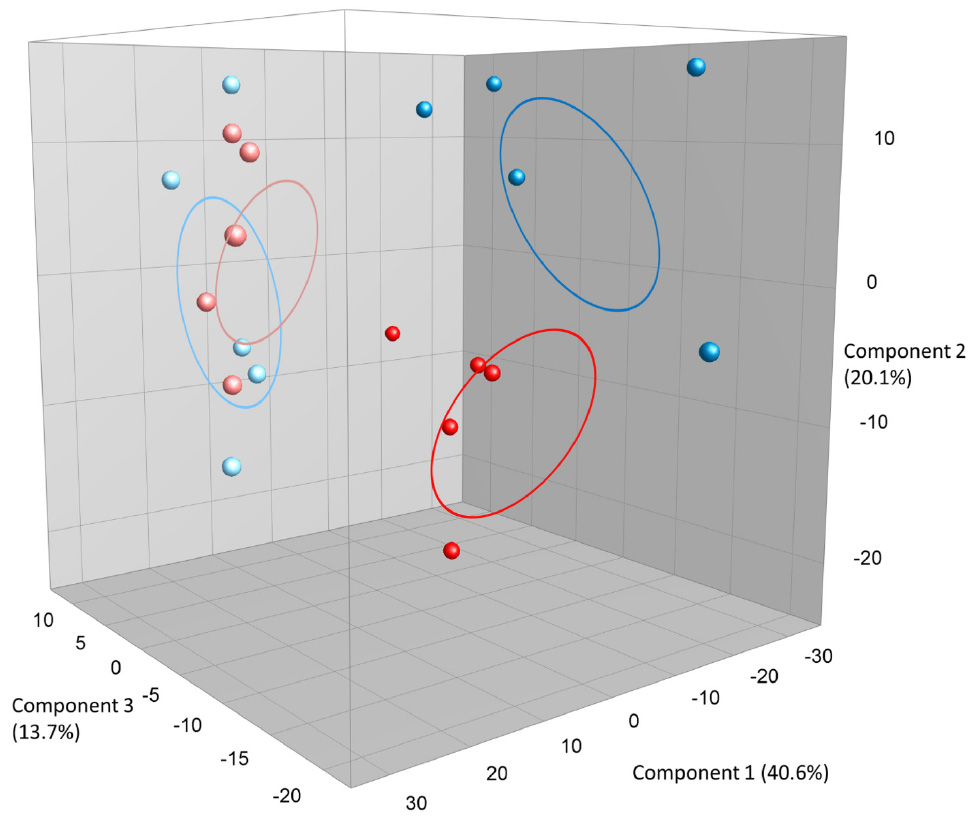
PCA plots based on DGGE profiles of 16S rRNA gene PCR-derived amplicons of BAL samples collected from six weeks old C57Bl/6 mice. The mice are both male and female offspring from C57Bl/6 dams exposed i.n. to either PBS or CNT particles. The colors represent red: CNT-male, pink CNT-female and dark blue PBS-male and light- blue PBS –female (n=20). DGGE: denaturing gradient gel electrophoresis after DGGE-PCR: polymerase chain reaction.

**Fig. (3a):**
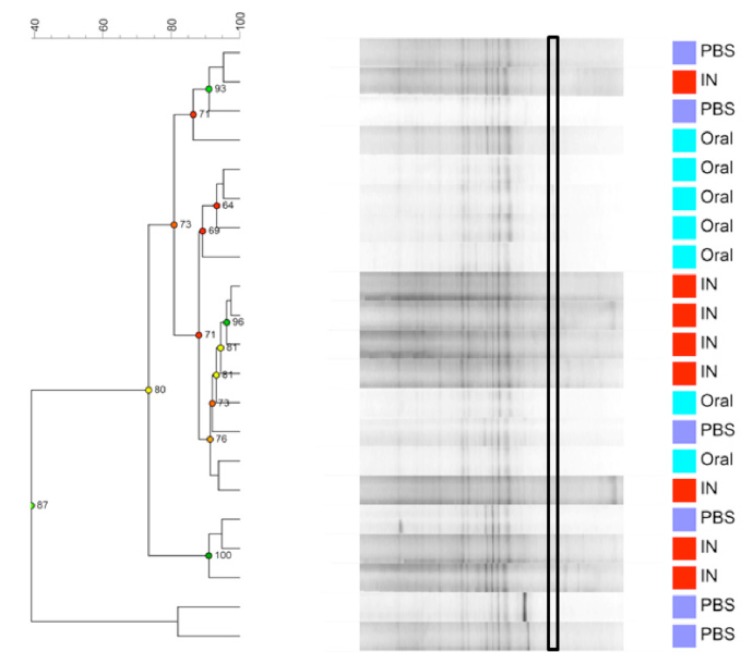
Dendrogram based on DGGE profiles representing 16S rRNA gene PCR-derived amplicons of caecum samples collected from eleven-week-old female BALB/cj mice. The light- blue (O) groups received oral exposure to Vancomycin from drinking water ad libitum. The red (IN) received vancomycin 0.21 mg/35µl nasally by i.n. 2x5 days. The blue PBS group received PBS 35µl nasally by i.n. 2x5 days. The band that separetes the O-group has the highest average intensity is marked with a black box. Reliably separated branches on the dendrogram have a high (green) cophenetic score.

**Fig. (3b):**
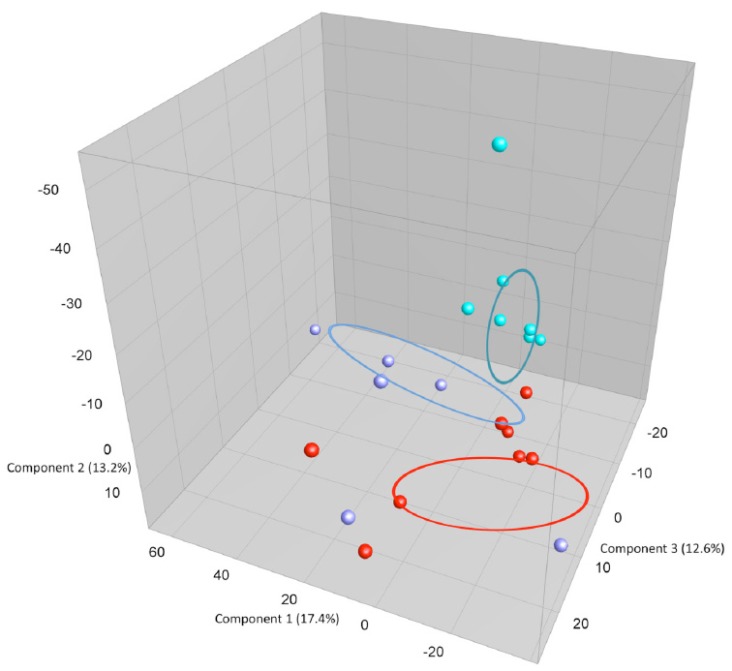
shows: PCA plots based on DGGE profiles of 16S rRNA gene PCR-derived amplicons of caecum samples collected from eleven-week-old female BALB/cj mice. The light-blue (O) groups received oral exposure to Vancomycin from drinking water ad libitum. The red (IN) received vancomycin 0.21 mg/35µl nasally by i.n.for 2x5 days. The blue PBS group received PBS 35µl nasally by i.n. 2x5 days. DGGE: denaturing gradient gel electrophoresis after DGGE-PCR: polymerase chain reaction.

**Fig. (3c):**
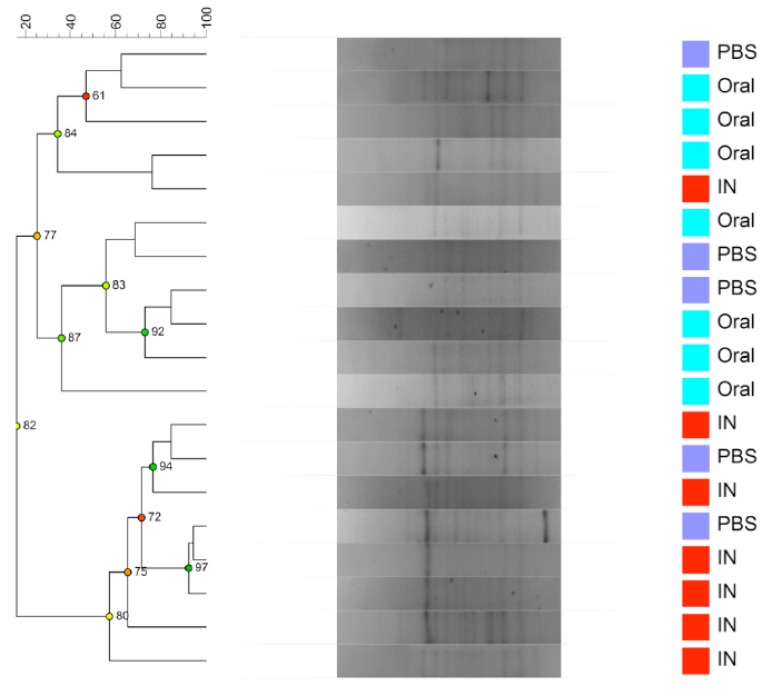
Dendrogram based on DGGE profiles representing 16S rRNA gene PCR-derived amplicons of BAL samples collected from eleven-week-old female BALB/cj mice. The light-blue (O) groups received oral exposure to Vancomycin from drinking water ad libitum. The red (IN) received vancomycin 0.21 mg/35µl nasally by i.n. 2x5 days. The blue PBS group received PBS 35µl nasally by i.n. 2x5 days. All IN Vancomycin exposed except one sample cluster together. Reliably separated branches on the dendrogram have a high (green) cophenetic score.

**Fig. (3d):**
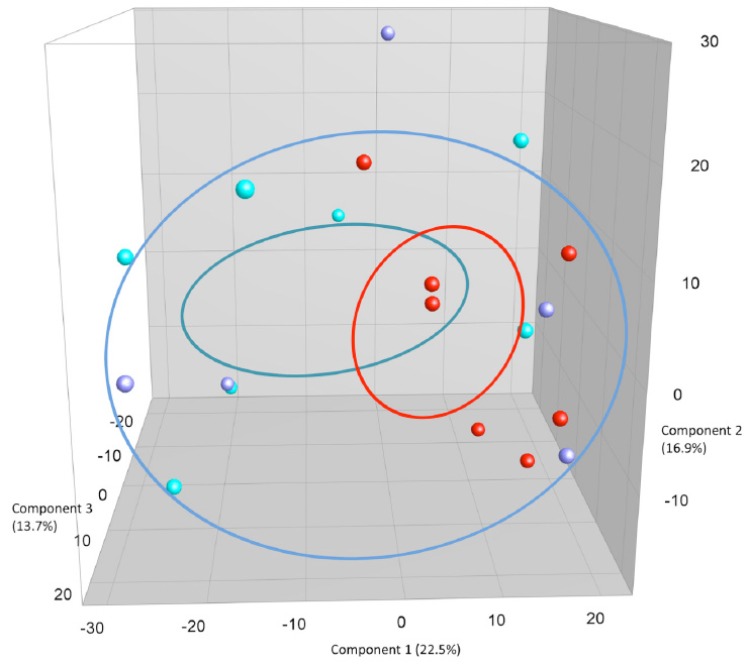
PCA plots based on DGGE profiles of 16S rRNA gene PCR-derived amplicons of BAL samples collected from eleven-week-old female BALB/cj mice. The light-blue (O) groups received oral exposure to vancomycin from drinking water ad libitum. The red (IN) received vancomycin 0.21 mg/35µl nasally by i.n. 2x5 days. The blue PBS group received PBS 35µl nasally by i.n. 2x5 days.
