# Supplementary material for: The Murine Lung Microbiome Changes During Lung Inflammation and Intranasal Vancomycin Treatment
Source: Open Microbiol J. 2015 Nov 3;9:167–79. doi: 10.2174/1874285801509010167 (PMC4676059; doi:10.2174/1874285801509010167)
Supplement: Supplementary file 1 [file TOMICROJ-9-167_SD1.pdf]

Open Access

Supplementary Material

The Murine Lung Microbiome Changes During Lung Inflammation and Intranasal Vancomycin Treatment

Kenneth Klingenberg Barfod[1,*], Katleen Vrankx[2], Hengameh Chloé Mirsepasi-Lauridsen[3], Jitka Stilund Hansen[1], Karin Sørig Hougaard[1], Søren Thor Larsen[1], Arthur C. Ouwenhand[4] and Karen Angeliki Krogfelt[3]

[1]National Research Centre for the Working Environment, Lersø parkallé 105, 2100 Denmark; [2]Applied Maths, Keistraat 120, 9830 Sint-Martens-Latem, Belgium; [3]Statens Serum Institut, Artillerivej 5, 2300 Denmark; [4]Active Nutrition, Dupont Nutrition & Health, Sokeritehtaantie 20, 02460 Kantvik Finland

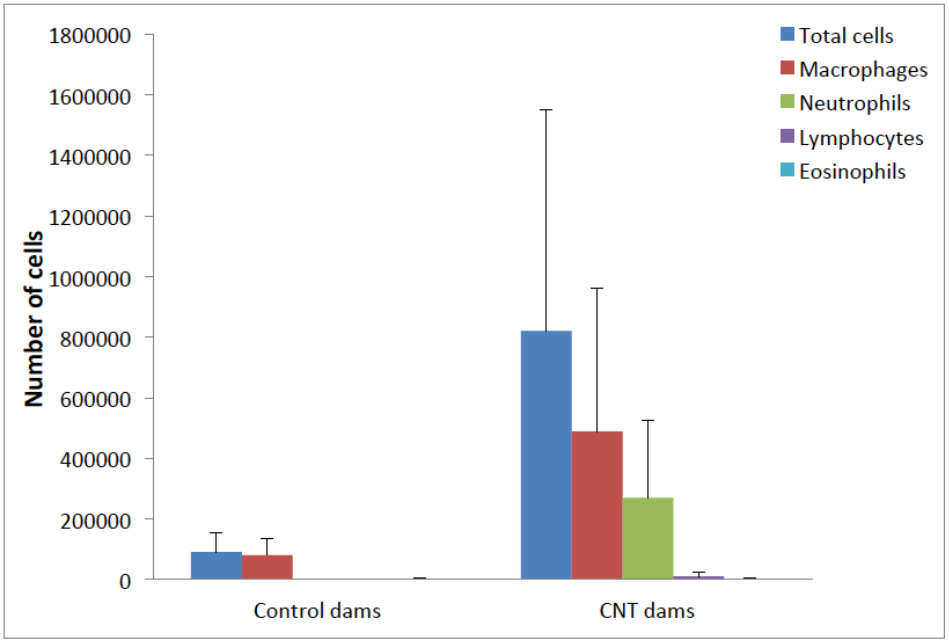

Supplementary Fig. (1). Airway inflammation in female dams 6 weeks following intratracheal instillation of PBS or 67 µg multiwalled carbon nanotubes (CNTs)(NM-400). Female mice were exposed to CNTs the day before mating. Lung lavage was performed 6 weeks later, i.e. shortly after weaning of pups. Mean and standard deviation of n=9-10 dams is shown. Statistically significant differences (examined by one-way ANOVA) were found between the two groups for the total number of cells ($p<0.001$), the number of macrophages ($p<0.001$), neutrophils ($p<0.001$), lymphocytes ($p<0.001$), but not for the number of eosinophils ($p=0.188$).
